# Association of Endothelial Nitric Oxide Synthase Polymorphisms with Clinical Severity in Patients with COVID-19

**DOI:** 10.3390/jcm14061931

**Published:** 2025-03-13

**Authors:** Aytekin İdikut, İlter Değer, Gamze Göktaş, Sevilay Karahan, Sevinç Sarınç, Deniz Köksal, Melih O. Babaoğlu, Elif Babaoğlu

**Affiliations:** 1 Department of Chest Diseases, Faculty of Medicine, Hacettepe University, Ankara 06230, Türkiyesevincsarinc@hacettepe.edu.tr (S.S.); deniz.koksal@hacettepe.edu.tr (D.K.);; 2 Department of Pharmacology, Faculty of Medicine, Hacettepe University, Ankara 06230, Türkiye; lter.deger@mku.edu.tr (İ.D.); babaoglu@hacettepe.edu.tr (M.O.B.); 3 Department of Bioistatistics, Faculty of Medicine, Hacettepe University, Ankara 06230, Türkiye; skarahan@hacettepe.edu.tr

**Keywords:** COVID-19 severity, endothelial dysfunction, genetic variants, NOS3, NOS3 polymorphisms, VNTR 4b/a

## Abstract

**Background/Objectives**: To elucidate the factors that contribute to individual variability in the progression of COVID-19, experiments on endothelial nitric oxide synthase polymorphisms have been reported. Nitric oxide synthase (NOS3) is located in the endothelium and is involved in the regulation of inflammation and vascular homeostasis. In this study, we investigated the association between COVID-19 severity and *NOS3 G894T* and *NOS3 27-bp VNTR 4b/a* genetic polymorphisms. **Methods**: Patients with COVID-19 (n = 178) were divided into Group 1 (mild disease) and Group 2 (severe disease) based on oxygen saturation levels in room air (Group 1, SpO_2_ ≥ 93%, n = 107; and Group 2, SpO_2_ < 93%, n = 73) and hospitalization requirements. Genotyping was performed using polymerase chain reaction-restriction fragment length polymorphism analysis. **Results**: Overall, genotype and allele frequencies of the *NOS3* genetic polymorphisms were similar across the two study groups (*p* > 0.05). However, the subgroup analysis showed a notable trend for the 4b/4a allele distribution between Groups 1 and 2. In the younger subgroup of patients (≤50 years old) without chronic obstructive pulmonary disease, Group 2 tended to have a higher frequency of the *4b* allele than Group 1 (97.4% vs. 85.4% *p* = 0.06) and a higher occurrence of 4b/4b genotype (94.7% vs. 74.0%, *p* = 0.05). Additionally, a rarely observed 4c allele was detected only in two subjects within Group 2 but not in Group 1. **Conclusions**: These findings suggest a trend of association between COVID-19 severity and *NOS3 27-bp VNTR 4b/a* genetic polymorphism. Genetic analysis may reveal patient susceptibility to disease, prognosis risk factors, and drug responsiveness.

## 1. Introduction

Coronavirus disease 2019 (COVID-19) is a respiratory illness caused by infection with severe acute respiratory syndrome coronavirus 2 (SARS-CoV-2). COVID-19 is extremely contagious, and its primary clinical manifestations include fever, dry cough, exhaustion, muscle soreness, and breathing difficulties. Moreover, dyspnea, hypoxemia, septic shock, or acute respiratory distress syndrome may develop approximately one week after the onset of the symptoms [1]. Given the extensive clinical range and inability to anticipate which individuals may experience severe symptoms, studies that may shed light on the factors influencing disease progression are needed.

Factors such as age, smoking habits, and the presence of chronic conditions, including chronic obstructive pulmonary disease (COPD), asthma, hypertension, and diabetes, might contribute to higher rates of illness and COVID-19-associated death [2,3]. Nevertheless, there is a lack of conclusive biomarkers that provide insights into disease progression. A comprehensive understanding of the genetic factors contributing to the severity of COVID-19 and the vulnerability to SARS-CoV-2 infection could enable the classification of individuals based on their risk level. This would enable the prioritization of immunization for high-risk individuals and contribute to the emergence of individualized treatment options [4]. Genome-wide association analysis may facilitate the identification of potential genetic factors implicated in the development of COVID-19. One study identified a gene cluster as a genetic risk locus in patients with COVID-19 experiencing respiratory failure and validated a possible role of the ABO blood group system [5]. Genetic association studies have demonstrated that genetic variations influencing SARS-CoV-2 receptors, such as angiotensin-converting enzymes or transmembrane serine protease-2 and immune components (interferons, interleukins, toll-like receptors, and human leukocyte antigen), are essential host determinants associated with the severity of COVID-19 [6,7]. Macrophage activation syndrome can result in septic shock, characterized by significant and potentially lethal systemic inflammation. However, the underlying risk factors and mechanisms contributing to the development of severe manifestations of COVID-19 have not yet been fully elucidated [8,9]. Patients with COVID-19 exhibit elevated blood levels of inflammatory markers, including C-reactive protein, lactate dehydrogenase (LDH), creatine phosphokinase (CPK), ferritin, D-dimer, and troponin-I, with a concomitant increase in the neutrophil-to-lymphocyte ratio and other inflammatory markers. Moreover, correlations were reported between elevated cytokine and chemokine levels and increased COVID-19 severity and mortality rates [10,11]. Therefore, studying the physiological pathways that may contribute to these inflammatory processes can help us understand disease development.

Nitric oxide (NO) is present in vascular tissues and blood cells and is primarily synthesized by endothelial NO synthase (NOS3). NO regulates platelet aggregation and smooth muscle cell contraction in blood vessels, helps mediate circulatory homeostasis, and regulates blood flow into tissues. Inhibition of platelet aggregation and leukocyte adhesion also suppresses inflammatory responses [12,13]. In addition, it increases the permeability from the circulation to tissues and alters the tone of vascular structures. Increased inflammation impairs the function of vital organs such as the lungs [14]. Variations in NO concentrations may alter the course of sepsis. Therefore, medications that alter NO concentrations could be administered in sepsis and other infectious diseases [15].

Polymorphisms in enzyme-coding genes may alter the activities of the corresponding enzymes. Variations in the *NOS3* gene, which may alter NOS3 enzyme expression or activity, have been shown to change the physiological functions of endothelium and blood cells, thereby contributing to disease progression [12,16,17,18]. *NOS3* genetic polymorphisms have been associated with various diseases, such as hypertension, chronic kidney disease, coronary artery disease, sickle cell anemia, subarachnoid hemorrhage, and diabetic retinopathy [19,20,21,22,23]. Furthermore, sepsis was associated with three *NOS3* genetic polymorphisms, *T(-786)C* (rs2070744); *27-bp VNTR 4b/a* (intron 4), and *G894T* (*Glu298Asp*, rs1799983). Recently, a study reported that the *NOS3 T(-786)C* rs2070744 genetic polymorphism was associated with susceptibility to develop sepsis among Turkish patients, potentially influencing the course of inflammation [14].

Recent investigations have examined the influence of genetic variations on the course of COVID-19. A meta-analysis revealed that polymorphisms in the ApoE, ACE1, TMPRSS2, CCR5, and HLA loci seem to influence susceptibility to and/or the severity of COVID-19 [24]. Additionally, a review provided a concise summary of the various genes that are linked to the severity of COVID-19 and are implicated in infection by SARS-CoV-2 [25]. Research on NOS3 gene polymorphisms has revealed an association between *NOS3* rs2070744 (T786C) and the severity of COVID-19 [26]. Further investigations are needed to elucidate factors contributing to individual variability in COVID-19 progression.

This study examined the association between COVID-19 severity and *NOS3* genetic variations. Our study analyzed the two NOS3 polymorphisms: *NOS3 G894T* (*Glu298Asp*) rs1799983 and *NOS3 27-bp VNTR 4b/a*.

## 2. Materials and Methods

### 2.1. Selection of Patients and Acquisition of Samples

**Figure 1:**
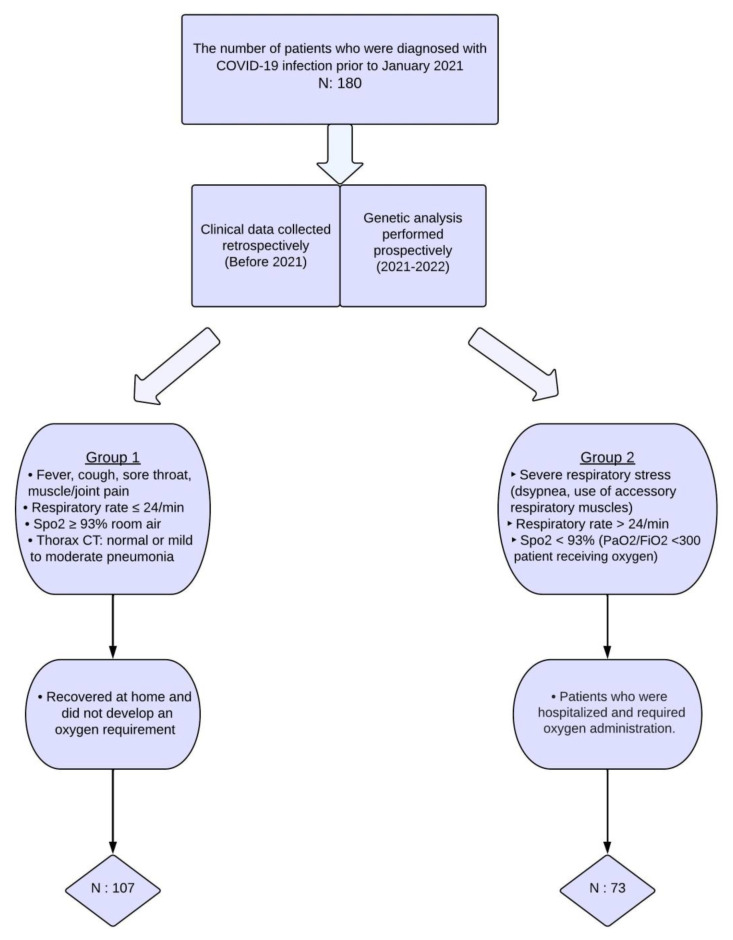
Selection process for inclusion of patients in the study.

A total of 180 subjects diagnosed with COVID-19 were recruited from the adult outpatient clinic of the Hacettepe University, Department of Chest Diseases, from December 2021 to September 2022. The participants were retrospectively categorized into two groups based on their disease course history during the COVID-19 pandemic. In order to conduct prospective genetic polymorphism analyses, venous blood samples were collected following the acquisition of written informed consent. The 107 patients who had mild disease comprised Group 1, whereas the 73 individuals with severe illness comprised Group 2. Figure 1 presents the criteria used to determine COVID-19 severity. Furthermore, the study did not include individuals who contracted the disease during the emergence of virus variants in 2021. Therefore, the study findings were not influenced by the impact of the viral variants on the disease course. This study also excluded environmental factors such as air pollution, seasonal variations, and the geographical locations of patients’ residences, as these were difficult to standardize and would further restrict patient selection.

Each study participant’s demographic and clinical information was documented in their case report form. Data on vital sign deterioration, critical care unit admission, and therapy administration interventions were collected. The date of COVID-19 diagnosis and the diagnostic method (polymerase chain reaction [PCR] or computed chest tomography) were documented for both groups. Additionally, we investigated whether pulmonary thromboembolism was detected during the disease course. In the retrospective analysis, smoking history and concurrent medical conditions were evaluated. The laboratory parameters (plasma levels of D-dimer, CRP, LDH, troponin-I, ferritin, and CPK, blood hemoglobin levels; the blood neutrophil, lymphocyte, and platelet counts, and the neutrophil/lymphocyte ratios) when COVID-19 was diagnosed were documented. The objective was to evaluate the prognosis of the disease by analyzing the laboratory parameters that were measured at the time of diagnosis prior to the commencement of treatment and follow-up procedures. All the data were collected during follow-up examinations and obtained from electronic medical records after obtaining written consent from the patients.

All procedures performed in this study involving human participants were in accordance with the ethical standards of the Helsinki Declaration, and the study was approved by the Hacettepe University Ethics Committee (Protocol Number: KA-21132). Informed consent was obtained from all patients included in the study.

### 2.2. Genotyping

Genomic DNA was purified from venous blood samples using a GeneJET Genomic DNA Purification Kit (Thermo Scientific, Waltham, MA, USA) according to the manufacturer’s instructions. Genotyping was performed by using a PCR-restriction fragment length polymorphism method for *NOS3 G894T* rs1799983 and a PCR method for *NOS3 27-bp VNTR 4b/a* genetic polymorphisms as described previously, with minor modifications [27].

A total volume of 25 or 50 μL containing 10 mM of dNTP, 1.5 mM of MgCl_2_, 20 mM of each primer, 5 U/μL of *Taq* DNA polymerase (Thermo Scientific, MA, USA), and 100 ng of genomic DNA was used as the PCR mixture. The PCR conditions were as follows: 95 °C for 5 min following 35 to 40 cycles of 95 °C and 56 °C to 61 °C for 1 min, and 72 °C for 5 to 10 min. The reactions were performed using a Applied Biosystems Thermal Cycler (Thermo Scientific, MA, USA). The amplified products for *NOS3 G894T* rs1799983 were restricted by 5 U/μL of BanII endonuclease (New England Biology Laboratories, Ipswich, MA, USA) at 37 °C overnight [19,21,27]. The primers used for DNA amplification, and the lengths of DNA products are summarized in Table 1. DNA fragments were separated by gel electrophoresis on 1% to 2% agarose gels and visualized under UV light by 0.5 to 1 μg/mL ethidium bromide staining (Kodak Gel Logic, New York, NY, USA).

### 2.3. Statistical Analyses

Categorical data, such as frequencies of genotypes and alleles among study groups, were compared using the chi-square test or Fisher’s exact test. The data were analyzed using Microsoft Excel 2019 (Microsoft Corporation, Redmond, WA, USA) and SPSS Version 23.0 software (IBM Corporation, Armonk, NY, USA). Where applicable, the distribution for the variables in the two study groups was compared using the independent Student’s *t*-test and Mann–Whitney *U* test. The significance was set at a *p*-value of <0.05.

## 3. Results

The patients’ demographic information, clinical characteristics, and comorbidities of the patients are summarized in Table 2. The mean ages of the patients in Groups 1 and 2 were 41.9 ± 14.6 and 60.2 ± 16.9 years old, respectively (*p* < 0.001). None of the patients exhibited pulmonary thromboembolism related to COVID-19.

As shown in Table 2, Group 2 comprised a significantly larger number of patients with type 2 diabetes mellitus, hypertension, coronary artery disease, heart failure, cardiac arrhythmia, COPD, and dementia than Group 1. At the time of diagnosis with COVID-19, the levels of D-dimer, CRP, LDH, troponin, ferritin, and neutrophil/lymphocyte ratios were significantly higher in Group 2 than those in Group 1; and hemoglobin levels were significantly lower in Group 2 (Table 2). No significant differences between the two groups were observed in the CPK levels and platelet and lymphocyte counts. The use of corticosteroids, enoxaparin, acetaminophen/NSAIDs, antibiotics, and low- and high-flow oxygen was significantly higher in Group 2, as also expected (Table 3).

For *NOS3 G894T* genetic polymorphism, the GG, GT, and TT genotype distributions were not significantly different between the groups (*p* = 0.85, Table 4). The wild-type G and the polymorphic T allele frequencies were also similar between the two groups (*p* = 0.78, Table 4).

The genotype and allele frequency distributions for *NOS3 27-bp VNTR 4b/a* genetic polymorphism between the study groups were similar (*p* = 0.44 and *p* = 0.28, respectively). Interestingly, for this genetic polymorphism, the ‘4c’ allele, previously reported in the literature as very rare, was observed in 2 of the 73 patients in Group 2. One patient carried the heterozygous 4b/4c genotype, and the other one the homozygous mutant 4c/4c genotype. Because of the very rare occurrence of the 4c allele, and as the frequency of genotypes and alleles containing the c allele in the study groups was insufficient to warrant a statistical evaluation, we analyzed the association between *NOS3 27-bp VNTR 4b/a* genetic polymorphism and the progression of COVID-19 by excluding these two subjects with the 4c allele.

Advanced age and COPD are known major confounding factors that may contribute to respiratory failure and worsen the progress of COVID-19. Therefore, we examined the distribution of genotypes and alleles in a subgroup of patients aged ≤50 years old and without COPD (n = 116). The patients with mild COVID-19 from this population were classified as “Subgroup 1” (n = 96), while those with severe illness were classified as “Subgroup 2” (n = 20) following our previous grouping. No significant differences were observed in distributions of the genotype (*p* = 0.68) or allele frequencies (*p* = 0.45) for *NOS3 G894T* genetic polymorphism between these two subgroups (Table 5).

Similarly, the genotype and allele distributions for the *NOS3 27-bp VNTR 4b/a* genetic polymorphism were similar between subgroups (*p* = 0.07, Table 5). However, there was a notable trend toward significance at the margin of the threshold because the 4b/4b genotype (*p* = 0.07) and the 4b allele (*p* = 0.06) were represented at a higher frequency in patients with severe COVID-19 than in those with mild disease (Table 5).

Upon categorizing the patients requiring hospitalization into two groups based on their requirement for intensive care, the duration of hospitalization for patients requiring intensive care was 5 days longer than that for patients who did not (*p* < 0.001). The mean age of the patients requiring intensive care group was significantly higher than that of the other groups (*p* = 0.009). No notable disparities were observed among the groups regarding sex, smoking history, or comorbidities. Statistically significant elevations were observed in the levels of LDH, troponin, and ferritin in the patients requiring intensive care compared to those who did not. In addition, patients who required intensive care were administered larger doses of corticosteroids, antibiotics, and acetaminophen/NSAIDs (Table S1).

The patients with severe COVID-19 who were administered high-flow oxygen or non-invasive mechanic ventilation treatment did not show any significant differences concerning age, sex, smoking history, or comorbidities compared with the group that received low-flow oxygen. Nevertheless, overall and intensive care unit stays were extended in the patients with severe COVID-19. Furthermore, the patients with severe COVID-19 had a greater requirement for corticosteroids, acetaminophen/NSAIDs, antibiotics, and vasopressors. Finally, only troponin levels were significantly higher in that group of patients (Table S2).

## 4. Discussion

COVID-19 continues to be a significant contributor to the global morbidity and mortality rates. This study aimed to evaluate the impact of genetic variants on disease outcomes, given that the clinical manifestations of COVID-19 have been linked to endothelial dysfunction and the role of NOS3 in vascular function.

Owing to individual variations in the progression of this disease, many studies have attempted to identify the factors that may influence inter-individual variability [1,2]. Diversity in disease progression among individuals is commonly attributed to comorbidities and patient age. Our study revealed that individuals with severe COVID-19 had a higher prevalence of coronary artery disease, heart failure, cardiac arrhythmias, hypertension, type-2 diabetes mellitus, and dementia, all of which are associated with advanced age (Table 2). The SARS-CoV-2 virus has the potential to induce cytokine cascades, which can have a detrimental impact on the circulatory system, resulting in morbidity and mortality. In diabetics, the development of COVID-19 disease is influenced by the upregulation of ACE-2, elevated levels of interleukin-6, and the impaired function of T cells. Atherosclerosis and hemodynamic instability may correlate with a more severe progression of COVID-19 in individuals with cardiovascular illnesses, maybe due to an elevation in inflammatory cytokines [1,2]. Host-related factors that may cause the progression or limitation of COVID-19 have been extensively studied. In severe cases, COVID-19 can cause respiratory and vascular diseases. Endothelial damage is an underlying factor in various COVID-19 complications [28,29,30]. This fact suggests that the endothelial cell barrier is breached in patients with severe disease progression. Although interferons are primarily involved in intracellular endothelial defense mechanisms, the levels of interferon-1 and -3 are low in patients with COVID-19 [30,31].

A study of 1936 participants revealed that obese patients with COVID-19 displayed a 1.34 times higher likelihood of being hospitalized compared to individuals with a normal body mass index [32]. Our investigation did not yield any statistically significant findings concerning the probability of obese patients being hospitalized or requiring oxygen support because of a COVID-19 diagnosis.

Biochemical and hematological parameters, such as neutrophil count, lymphocyte count, neutrophil/lymphocyte ratio, CRP, interleukin-6, D-dimer, troponin-I, ferritin, and creatine kinase, were investigated as prognostic indicators for individuals with COVID-19. Biochemical values like D-dimer, CRP, and ferritin serve as important indicators of enhanced systemic inflammation and endothelial dysfunction [10,11,33]. We found that the levels of D-dimer, CRP, LDH, ferritin, and troponin-I, as well as the neutrophil-to-lymphocyte ratio, were elevated in patients with severe disease (Group 2) as compared to those in patients with mild disease (Group 1). The levels of LDH, troponin-I, and ferritin were higher in the patients who required intensive care than those who required hospitalization without intensive care treatment.

Nitric oxide (NO) is an important autacoid responsible for the homeostasis of many human body functions. Among its diverse actions in the body, antiviral and anti-inflammatory properties may be important in infections and inflammatory disorders. A study conducted after the SARS outbreak in 2005 revealed that NO synthesized by iNOS inhibited the SARS-CoV replication cycle and reduced RNA and protein synthesis [34]. Increased mortality rates in diseases with underlying pathophysiological endothelial damage suggest that NO produced by NOS3 plays an important protective role [35].

Risk factors for COVID-19, such as advanced age, hypertension, and diabetes, are also associated with endothelial dysfunction [36]. NO inhalation treatment in patients with acute respiratory distress syndrome provides beneficial effects, but its contribution to mortality could not be determined [37]. In addition, elevated NO levels produced by NOS3 were reported during inflammation [15]. Moreover, in infective processes involving endothelial cells, NOS3-mediated NO production prevents the proliferation of bacteria, such as *Mycobacterium tuberculosis*. This supports the idea that the endothelial structure and NOS3 play important modulatory roles in the immune system [35,38]. In the endothelium, NOS3 has a secretory effect on erythropoietin, which protects children and young adults from COVID-19 [39]. The *NOS3* genetic polymorphisms *T(-786)C*, *27-bp VNTR 4b/a*, and *G894T* influence the NOS3 expression and/or activity; thus, considering the NOS3 role in the immune system defense against COVID-19, genetic polymorphisms may be associated with the course and severity of COVID-19 [12,14,19,20,21,22,23,40,41].

In a previous study examining the impact of *NOS3* genetic polymorphisms on NOS3 function in blood cells of healthy, non-smoking individuals, an increase in erythrocyte aggregation index was reported in individuals carrying the 4b/4b genotype for *NOS3 27-bp VNTR 4b/a* polymorphism. In contrast, a decrease in aggregation was observed in individuals carrying the TT genotype for *NOS3 G894T* genetic polymorphism [14]. Genetic variations in *NOS3* may contribute to the development of microvascular disorders by interfering with erythrocyte aggregation and deformability [12,14].

Changes in the number of repeats of a 27-bp sequence in the fourth intronic region of the *NOS3* gene (*27-bp VNTR 4b/a*) reduce the synthesis of the NOS3 enzyme [42]. Previous studies have reported an association of this polymorphism with endothelial dysfunction due to the decreased NOS3 activity in patients with early-onset primary hypertension, diabetes mellitus, and subarachnoid hemorrhage [43,44,45]. A meta-analysis by Shi et al. (2021) revealed a significant relationship between the *NOS3* G894T genetic polymorphism and the risk of developing hypertension [23]. Another meta-analysis could not determine an association between diabetic retinopathy in patients with type 2 diabetes mellitus and *NOS3 27-bp VNTR 4b/a* genetic polymorphism [21]. The *NOS3 G894T* genetic polymorphism was also associated with preeclampsia susceptibility in pregnant Turkish patients [46]. Similarly, *NOS3 T(-786)C* and *NOS3 27-bp VNTR 4b/a* genetic polymorphisms were associated with the risk of developing preeclampsia [47]. A study conducted in Turkish patients who were followed up in the emergency department for sepsis has shown that the *NOS3 T(-786)C* genetic polymorphism might increase the susceptibility to sepsis [14]. In our study, no statistically significant associations were detected between the genotype and allele frequencies of the *NOS3 G894T* or *NOS3 27-bp VNTR 4b/a* genetic polymorphisms and COVID-19 severity. The absence of this association may have been due to the multifactorial nature of COVID-19 progression, sample size limitations that interfered with statistical power, or the presence of other *NOS3* gene polymorphisms or genes that might affect the endothelium. We found a trend in the association of disease severity and patient genotypes at the margin of statistical significance level in a subgroup of younger patients (<50 years of age) without COPD (see Table 5). The rationale for comparing genetic analyses in individuals under 50 years of age is that this demographic typically exhibits a lower prevalence of systemic diseases, thereby minimizing the impact of comorbidities on disease prognosis and rendering genetic predisposition a more significant determinant of COVID-19 outcomes.

In a study of the *NOS3 27-bp VNTR 4b/a* genotypes of patients with asthma in the United States, the three alleles, 4a, 4b, and 4c, were observed among non-African-American patients with asthma. In contrast, only 4a and 4b alleles were observed in the control group. That study proposed that the uncommon 4c allele could be a risk factor for individuals with asthma [48]. Interestingly, in our study, the very rare 4c allele was detected only in two patients with asthma who experienced severe COVID-19. Although the 4c/4c genotype was previously identified in a study including healthy adults [49], our study detected, for the first time, the 4c/4c polymorphic homozygous genotype in a patient with asthma.

In a meta-analysis examining the frequency of bacterial infections accompanying COVID-19 and antibiotic treatment, 1058 studies were evaluated, and antibiotic use in 76,176 patients with COVID-19 was investigated. Although the occurrence of concurrent bacterial infections was 5.62%, a significant proportion of patients (61.77%) received antibiotic therapy. Therefore, unnecessarily large amounts of antibiotics were used despite low co-infection rates [50]. In our study, more antibiotics were administered to hospitalized patients with severe COVID-19 than to those with mild illness. Antibiotic use was more common in patients who were monitored in intensive care units and in those requiring high-flow oxygen therapy. This could be attributed to the fact that hospital-acquired infections may accompany COVID-19 or because antibiotics are administered to avoid possible bacterial infections in hospitalized patients. Excessive antibiotic use in viral infections can contribute to resistance. Improved diagnostic tools are necessary to distinguish bacterial co-infections from viral inflammation, thereby preventing unnecessary antibiotic administration.

### Limitations

Because NO levels were not quantified in our research, an inherent limitation of our investigation was the inability to analyze blood NO levels in relation to gene polymorphisms. Confounding factors such as regular medication use or lifestyle were not examined in our study. Participants could not be evenly categorized by age and gender due to an inadequate sample size to identify patients who fulfilled the inclusion criteria. The influence of age on the illness process could not be entirely disregarded due to this condition. The lack of statistically significant results in our study may be attributed to factors such as the different ethnic backgrounds of the participants compared to other studies and insufficient sample size. Moreover, the number of participants must be increased to achieve more statistically significant results.

## 5. Conclusions

We found a trend for an association of *NOS3 27-bp VNTR 4b/a* genetic polymorphism with the disease course of COVID-19. Briefly, a tendency for higher frequencies of the homozygous wild-type *4b/4b* genotype and *4b* allele was detected in patients with severe COVID-19 than in those with mild disease in a subgroup of patients without COPD who were under 50 years of age. Predictive markers are needed to determine the disease course for COVID-19. Pharmacogenomic studies are becoming increasingly important for the early diagnosis and prognostic determination of diseases, including COVID-19. Genetic studies on endothelial dysfunction and inflammation may have pharmacogenomic implications, warranting further research on the potential role of NOS3 in COVID-19 treatment strategies.

## Figures and Tables

**Table 1:** Primers used for PCR and lengths of products.

Genetic Polymorphism	PCR ^a^ Primers	PCR Product Length (bp)	Restriction Enzyme	Restriction Fragment Length (bp)	Allele
*NOS3 G894T* rs1799983	5′–AAGGCAGGAGACAGTGGATGGA–3′	248	BanII	163 + 85	G
	5′–CCCAGTCAATCCCTTTGGTGCTCA–3′			248	T
*NOS3 27-bp* *VNTR 4b/a*	5′–AGGCCCTATGGTAGTGCCTTT–3′	*No restriction. The lengths of PCR products for 4a, 4b, and 4c alleles are 447, 420, and 393 bp, respectively.*			
	5′–TCTCTTAGTGCTGTGGTCAC–3′				

^a^ Polymerase chain reaction.

**Table 2:** Demographic information, clinical characteristics, and comorbidities of the patient groups.

	Group 1 (Mild) (n = 107)	Group 2 (Severe) (n = 73)	*p*-Value
Age ^a^ (years)	41.9 ± 14.7	60.2 ± 16.9	**<0.001**
Sex (n) (%) Female ^b^ Male ^b^			
	75 (70.1%)	37 (50.7%)	**0.008**
	32 (29.9%)	36 (49.3%)	
Cigarettes ^b^ (box/years)	6.9 ± 11.9	10.8 ± 15.6	0.114
Diagnostic method (n) (%) PCR test ^b^ Thorax CT (PCR negative) ^b^			
	105 (98.1%)	71 (97.3%)	
	2 (1.9%)	2 (2.7%)	1.000
Pulmonary embolism (COVID-19 related) ^b^	0	0	
Comorbidities (n) (%)			
Type 2 diabetes ^b^	19 (17.8%)	25 (34.2%)	**0.011**
Hypertension ^b^	23 (21.5%)	28 (38.4%)	**0.014**
Coronary artery disease ^b^	5 (4.7%)	18 (24.7%)	**<0.001**
Heart failure ^b^	7 (6.5%)	22 (30.1%)	**<0.001**
Cardiac arrhythmia ^b^	2 (1.9%)	9 (12.3%)	**0.008**
COPD ^b^	6 (5.6%)	14 (19.2%)	**0.009**
Asthma ^b^	15 (14.0%)	14 (19.2%)	0.473
GI diseases ^b^	8 (7.5%)	1 (1.4%)	0.086
Liver diseases ^b^	3 (2.8%)	2 (2.7%)	0.980
Kidney diseases ^b^	3 (2.8%)	6 (8.2%)	0.161
Hyperthyroidism ^b^	1 (0.9%)	0	
Hypothyroidism ^b^	13 (12.1%)	14 (19.2%)	0.278
Hyperlipidemia ^b^	5 (4.7%)	5 (6.8%)	0.768
Connective tissue disease ^b^	5 (4.7%)	6 (8.2%)	0.510
Dementia ^b^	3 (2.8%)	14 (19.2%)	**<0.001**
Obesity ^b^	9 (8.4%)	13 (17.8%)	0.097

^a^ Continuous variables with normal distribution are presented as means ± standard deviations. ^b^ Categorical variables are presented as numbers and percentages. GI disease: Ulcerative colitis, Crohn’s disease, peptic ulcer. Kidney disease: Kidney cysts or nephrotic syndrome causing mild kidney failure.

**Table 3:** Comparison based on prognostic laboratory results and treatments received.

	Group-1 (n = 107)	Group-2 (n = 73)	*p*-Value
D-dimer ^a^ (mg/L)	0.55 ± 0.40	1.86 ± 2.97	**0.001**
C-reactive protein ^a^ (mg/dL)	1.48 ± 1.59	14.16 ± 23.49	**0.001**
Lactate dehydrogenase ^a^ (U/L)	166.79 ± 59.42	316.90 ± 148.58	**0.001**
Troponin-I ^a^ (ng/L)	4.94 ± 5.16	147.06 ± 775.47	**0.024**
Ferritin ^a^ (µg/L)	101.0 ± 84.1	273.1 ± 351.5	**0.004**
Creatine phosphokinase ^a^ (U/L)	112.8 ± 142.8	151.5 ± 159.1	0.275
Hemoglobin ^a^ (g/dL)	13.9 ± 1.3	12.8 ± 1.9	**0.001**
Platelet count ^a^ (10^3^/µL)	248.3 ± 94.9	222.6 ± 113.5	0.221
Lymphocyte numbers ^a^ (10^3^/µL)	1.643 ± 0.6	1.296 ± 1.2	0.092
Neutrophil/lymphocyte ratio ^b^	3.05 (2.26–3.59)	4.45 (2.58–6.18)	**0.001**
DRUGS ADMINISTERED			
Corticosteroid ^c^	6 (5.6%)	58 (79.5%)	**0.001**
Enoxaparin ^c^	13 (12.1%)	69 (94.5%)	**0.001**
Tocilizumab	0	0	
Anakinra	0	2 (2.7%)	
Favipiravir ^c^	42 (39.3%)	37 (50.7%)	0.129
Remdesivir	0	1 (1.4%)	
Hydroxychloroquine ^c^	6 (5.6%)	8 (11.0%)	0.302
IVIG	0	3 (4.1%)	
Plasma exchange	0	3 (4.1%)	
Paracetamol/NSAIDs ^c^	50 (46.7%)	52 (71.2%)	**0.002**
Acetylsalicylic acid ^c^	23 (21.5%)	18 (24.7%)	0.752
Antibiotic use ^c^	4 (3.7%)	34 (46.6%)	**0.001**
Low-flow oxygen ^c^	0	73 (100%)	**0.001**
High-flow oxygen ^c^	0	23 (31.5%)	**0.001**
NIMV ^c^	0	18 (24.7%)	**0.001**
Vasopressor	0	4 (5.5%)	

^a^ Continuous variables with normal distribution are presented as means ± standard deviations. ^b^ Non-normally distributed continuous variables are presented using their median, minimum, and maximum values. ^c^ Categorical variables are presented using numbers and percentages. IVIG, intravenous immunoglobulin; NIMV, non-invasive mechanical ventilation; NSAIDs, non-steroidal anti-inflammatory drugs.

**Table 4:** Genotype and allele frequencies of *NOS3 G894T* rs1799983 and *NOS3 27-bp VNTR 4b/a* genetic polymorphisms.

Genetic Polymorphism	Group 1 (n = 107) (Patients with Mild COVID-19)		Group 2 (n = 73) Patients with Severe COVID-19		*p*-Value; (χ^2^, df)
***NOS3 G894T* rs1799983**	n	(%)	n	(%)	
Genotypes					
GG	55	(51.4)	40	(54.8)	0.85; (0.31, 2)
GT	38	(35.5)	23	(31.5)	
TT	14	(13.1)	10	(13.7)	
Alleles					
G	148	(69.2)	103	(70.5)	0.78; (0.08, 1)
T	66	(30.8)	43	(29.5)	
***NOS3 27-bp VNTR 4b/a***					
Genotypes					
4b/4b	79	(73.8)	56	(78.9)	0.44; (0.59, 1)
4b/4a and 4a/4a	28	(26.2)	15	(21.1)	
Alleles					
4b	183	(85.5)	127	(89.4)	0.28; (1.17, 1)
4a	31	(14.5)	15	(10.6)	

**Table 5:** Genotype and allele frequencies of *NOS3 G894T* rs1799983 and *NOS3 27-bp VNTR 4b/a* genetic polymorphisms among patients who did not have COPD and were ≤50 years of age.

Genetic Polymorphism	Subgroup 1 (n = 96) (Patients with Mild COVID-19)		Subgroup 2 (n = 20) (Patients with Severe COVID-19)		*p*-Value; (χ^2^, df)
***NOS3 G894T* rs1799983**	n	(%)	n	(%)	
Genotypes					
GG	57	(59.4)	11	(55.0)	0.68; (0.79, 2)
GT	27	(28.1)	5	(25.0)	
TT	12	(12.5)	4	(20.0)	
Alleles					
G	141	(73.4)	27	(67.5)	0.45; (0.58, 1)
T	51	(26.6)	13	(32.5)	
***NOS3 27-bp VNTR 4b/a***					*p*-value (Fisher’s exact test)
Genotypes					
4b/4b	71	(74.0)	18	(94.7)	0.07
4b/4a and 4a/4a	25	(26.0)	1	(5.3)	
Alleles					
4b	164	(85.4)	37	(97.4)	0.06
4a	28	(14.6)	1	(2.6)	

## Data Availability

The datasets supporting the findings of the current study are openly available in the Zenodo repository at https://zenodo.org/records/14063783, accessed on 12 January 2025.

## Supplementary Materials

The following supporting information can be downloaded at: https://www.mdpi.com/article/10.3390/jcm14061931/s1, Table S1: Hospitalization time, laboratory values, and drugs used in patients requiring intensive care versus those not requiring intensive care; Table S2: Hospitalization durations, treatment information, and laboratory results of patients receiving low-flow oxygen therapy and patients requiring high-flow oxygen or non-invasive mechanical ventilator therapy.

## Abbreviations

The following abbreviations are used in this manuscript:

ACE-2	Angiotensin-Converting Enzyme-2
COPD	chronic obstructive pulmonary disease
COVID-19	coronavirus disease 2019
NOS3	endothelial nitric oxide synthase
IVIG	intravenous immunoglobulin
LDH	lactate dehydrogenase
NIMV	non-invasive mechanical ventilation
NO	nitric oxide
PCR	polymerase chain reaction
SARS-CoV-2	severe acute respiratory syndrome coronavirus 2
