# Supplementary material for: Association of Endothelial Nitric Oxide Synthase Polymorphisms with Clinical Severity in Patients with COVID-19
Source: J Clin Med. 2025 Mar 13;14(6):1931. doi: 10.3390/jcm14061931 (PMC11943162; doi:10.3390/jcm14061931)
Supplement: Supplementary file 1 [file jcm-14-01931-s001.zip › SUPPLEMENTARY_TABLE_S1.pdf]

Table S1. Hospitalization time, laboratory values, and drugs used in patients requiring intensive care versus those not requiring intensive care

	**Patients requiring intensive care** (n=36)	**Patients not requiring intensive care** (n=37)	***p*-value**
**Total hospitalization time**	14.0 (6.5–13.5)	9.0 (11.25–30)	**0.001**
**D-dimer (mg/L)** [a]	1.155 (0.56–2.1)	0.86 (0.39–1.62)	0.133
**C-reactive protein (mg/dL)** [a]	7.875 (3.32–15.32)	4.45 (0.9–16.71)	0.132
**Lactate dehydrogenase (U/L)** [b]	361.97 ± 185.43	274.74 ± 86.75	**0.026**
**Troponin-I (ng/L)** [a]	8.5 (4.37–19)	3.4 (0.825–7.25)	**0.004**
**Ferritin (µg/L)** [a]	171.5 (75.35–526.97)	141 (33.3–238)	**0.042**
**Creatine phosphokinase (U/L)** [a]	111 (40.5–204)	93 (67–175)	0.858
**Hemoglobin (g/dl)** [b]	12.64 ± 2.15	13.04 ± 1.83	0.433
**Platelet (10^3/µL)** [b]	223.5 ± 133.26	221.6 ± 91.14	0.947
**Lymphocyte (10^3/µL)** [b]	1.291 ± 1.602	1.301 ± 0.716	0.975
**Neutrophil/lymphocyte ratio** [a]	4.519 (2.72–11.04)	4.34 (2.45–5.27)	0.234
**Corticosteroid** [c]	34 (94.4%)	24 (64.8%)	**0.005**
**Enoxaparin** [c]	33 (91.6%)	36 (97.2%)	0.358
**Favipiravir** [c]	20 (55.5%)	17 (45.9%)	0.557
**Hydroxychloroquine** [c]	4 (11.1%)	4 (10.8%)	1.000
**Acetaminophen/NSAIDs** [c]	32 (88.8%)	20 (54.0%)	**0.002**
**Acetylsalicylic acid** [c]	10 (27.7%)	8 (21.6%)	0.735
**Antibiotic use** [c]	23 (63.8%)	11 (29.7%)	**0.007**

[a] Continuous variables with normal distribution are presented as means ± standard deviations.

[b] Non-normally distributed continuous variables are presented using their median and minimum and maximum values.

[c] Categorical variables are presented using numbers and percentages.

NSAIDs, non-steroidal anti-inflammatory drugs
