# Supplementary material for: Association of Endothelial Nitric Oxide Synthase Polymorphisms with Clinical Severity in Patients with COVID-19
Source: J Clin Med. 2025 Mar 13;14(6):1931. doi: 10.3390/jcm14061931 (PMC11943162; doi:10.3390/jcm14061931)
Supplement: Supplementary file 1 [file jcm-14-01931-s001.zip › SUPPLEMENTARY_TABLE_S2.pdf]

Table S2. Hospitalization durations, treatment information, and laboratory results of patients receiving low-flow oxygen therapy and patients requiring high-flow oxygen or non-invasive mechanical ventilator therapy

	**Low-flow oxygen therapy** n=44	**High-flow oxygen/ Non-invasive mechanical ventilator therapy** n=29	***p*-value**
**Total hospitalization time**	10.0 (7–13)	20.0 (12–40)	**<0.001**
**Intensive care stay**	0.0 (0–0)	12.0 (5–12)	**<0.001**
**Corticosteroid** [b]	29 (65.9%)	29 (100%)	**0.001**
**Enoxaparin** [b]	41 (93.18%)	28 (96.55%)	1.000
**Favipiravir** [b]	20 (45.45%)	17 (58.62%)	0.389
**Hydroxychloroquine** [b]	5 (11.36%)	3 (10.34%)	1.000
**Acetaminophen/NSAIDs** [b]	26 (59.09%)	26 (89.65%)	**<0.011**
**Acetylsalicylic acid** [b]	9 (20.45%)	9 (31.03%)	0.454
**Antibiotic use** [b]	14 (31.81%)	20 (68.96%)	**<0.004**
**Vasopressor** [b]	0	4 (13.79%)	0.022
**D-dimer (mg/L)** [a]	0.94 (0.41–1.77)	1.00 (0.43–1.99)	0.660
**C-reactive protein (mg/dL)** [a]	5.62 (1.12–16.8)	6.97 (3.68–13.57)	0.247
**Lactate dehydrogenase (U/L)** [c]	284.11 ± 93.55	366.08 ± 197.76	0.068
**Troponin-I (ng/L)** [a]	4.35 (0.87–8.15)	9.15 (4.51–19.6)	**<0.007**
**Ferritin (μg/L)** [a]	150.5 (54.12–26)	175.0 (81.5–415.0)	0.197
**Creatine phosphokinase (U/L)** [a]	85.5 (41.75–173.2)	113.5 (59.0–204.5)	0.328
**Hemoglobin (g/dL)** [c]	12.98 ± 1.71	12.63 ± 2.35	0.516
**Platelet (10^3/μL)** [c]	232.62 ±118.76	208.38 ± 106.4	0.409
**Lymphocyte (10^3/μL)** [c]	1.25 ± 0.696	1.35 ± 1.759	0.751
**Neutrophil/lymphocyte ratio** [a]	4.34 (2.67–5.56)	4.53 ± (2.57–11.43)	0.375

[a] Continuous variables with normal distribution are presented as means ± standard deviations.

[b] Non-normally distributed continuous variables are presented by their median value and minimum and maximum values.

[c] Categorical variables are presented using numbers and percentages.

NSAIDs, non-steroidal anti-inflammatory drugs
